# Partial Surface Modification of Low Generation Polyamidoamine Dendrimers: Gaining Insight into their Potential for Improved Carboplatin Delivery

**DOI:** 10.3390/biom9060214

**Published:** 2019-06-02

**Authors:** Dai Hai Nguyen, Long Giang Bach, Diem-Huong Nguyen Tran, Van Du Cao, Thi Nhu Quynh Nguyen, Thi Thu Hong Le, Thach Thao Tran, Thai Thanh Hoang Thi

**Affiliations:** 1 Institute of Applied Materials Science, Vietnam Academy of Science and Technology, Ho Chi Minh City 700000, Vietnam; nguyendaihai@iams.vast.vn (D.H.N.); trannguyendiemhuongbiotech@gmail.com (D.-H.N.T.); 2 Graduate University of Science and Technology, Vietnam Academy of Science and Technology, Hanoi 100000, Vietnam; 3 Nguyen Tat Thanh University, 300A Nguyen Tat Thanh, District 4, Ho Chi Minh City 700000, Vietnam; blgiang@ntt.edu.vn; 4 Faculty of Pharmacy, Lac Hong University, Buu Long Ward, Bien Hoa City, Dong Nai Province 810000, Vietnam; caovandulhu@gmail.com (V.D.C.); ds.nhuquynhnguyen@gmail.com (T.N.Q.N.); Hongle5792@gmail.com (T.T.H.L.); tranthachthao185@gmail.com (T.T.T.); 5 Biomaterials and Nanotechnology Research Group, Faculty of Applied Sciences, Ton Duc Thang University, Ho Chi Minh City 700000, Vietnam

**Keywords:** carboplatin, cancer treatment, low generation polyamidoamine (PAMAM) dendrimer, surface modification, drug delivery systems

## Abstract

Carboplatin (CAR) is a second generation platinum-based compound emerging as one of the most widely used anticancer drugs to treat a variety of tumors. In an attempt to address its dose-limiting toxicity and fast renal clearance, several delivery systems (DDSs) have been developed for CAR. However, unsuitable size range and low loading capacity may limit their potential applications. In this study, PAMAM G3.0 dendrimer was prepared and partially surface modified with methoxypolyethylene glycol (mPEG) for the delivery of CAR. The CAR/PAMAM G3.0@mPEG was successfully obtained with a desirable size range and high entrapment efficiency, improving the limitations of previous CAR-loaded DDSs. Cytocompatibility of PAMAM G3.0@mPEG was also examined, indicating that the system could be safely used. Notably, an in vitro release test and cell viability assays against HeLa, A549, and MCF7 cell lines indicated that CAR/PAMAM G3.0@mPEG could provide a sustained release of CAR while fully retaining its bioactivity to suppress the proliferation of cancer cells. These obtained results provide insights into the potential of PAMAM G3.0@mPEG dendrimer as an efficient delivery system for the delivery of a drug that has strong side effects and fast renal clearance like CAR, which could be a promising approach for cancer treatment.

## 1. Introduction

Platinum-based chemotherapeutics have become the mainstay of modern oncology by being one of the most extensively prescribed drugs, either alone or in combination with other drugs. Nearly 50% of all cancer patients undergoing chemotherapy were given a platinum-based drug. As the second generation platinum (II) anticancer compound, carboplatin (CAR) gained Food and Drug Administration (FDA) approval in 1989 and has received increasing attention in recent years [1,2,3]. It has been shown to be very effective in the treatment of ovarian, lung, bladder, head and neck, esophageal, endometrial cancer, and so forth. [4,5]. Owning to the bidentate dicarboxylate leaving group instead of more labile chlorides, CAR has significantly reduced side effects relative to the first generation platinum-based drug cisplatin and presented as effective on several types of cancer that are not susceptible to cisplatin [1]. Unfortunately, its clinical use is restricted due to non-specific distribution within the body, leading to dose-limiting toxicity bone marrow suppression, particularly thrombocytopenia and anemia [6]. As reported by Zalba et al., 90% of erythrocytes could be lost compared to the normal level [7]. Besides, fast renal clearance with 90% of administered CAR being excreted within 24 h is another drawback of CAR [8]. Some drug delivery systems (DDS) such as liposomes [9], carbon-based nanocontainers [10] and polycaprolactone (PCL) nanoparticles [11] were developed for CAR delivery to address its drawbacks; however, an undesirable size range (> 200 nm or even up to micrometer scale) and low CAR loading efficiency have remained as these systems’ limitation. 

Dendrimer, especially polyamidoamine (PAMAM) dendrimer, turned out to be one of the most ideal DDS for therapeutic agents [12]. As for its important roles, PAMAM dendrimers were well characterized and commercialized under the trade name of Starburst® Dendrimer owned by Starpharma, Melbourne, Australia. Not only this one but also a lot of another PAMAMs were developed variously by Dendritech®, Milwaukee, WI, USA; by Dendritic Nanotechnologies, Mt. Pleasant, MI, USA; recently by NanoSynthons, LLC, Mt. Pleasant, MI, USA; and so forth [13]. This approach was very convenient for many researchers/clinicians to study on novel therapeutic/diagnostic agent encapsulated into PAMAM. This is thanks to the highly branched, radial and stable structure of PAMAM dendrimer that facilitates the host-guest entrapment property. Drug molecules can be entrapped on the surface within their large internal cavities, or interspersed throughout the dendritic structure, thus protecting them from physiological degradation [3,14,15,16]. Many studies have reported that loaded anticancer drugs (5-fluorouracil, methotrexate, paclitaxel, camptothecin, 6-mercaptopurin, and doxorubicin) in PAMAM dendrimer exhibited enhanced stability, anticancer activity and reduced side effects [3,17,18]. Their highly controllable size in a range of 1–100 nm also endowed the passive targeting of the drug to the tumor site via enhanced permeation and retention (EPR) effect, thus improving the anticancer activity and reducing the side effects of drug to normal cells [19,20,21]. Looking at the commercial products of substance-loaded dendrimers for biomedical applications within a time interval of 2000–2010, there were many outcomes for many fields but not for anti-cancer areas. VivaGel® (Starpharma, Melbourne, Australia) is an antiviral dendrimer-based topical pharmaceutical approved by the U.S Food and Drug Administration (FDA). SuperFect® Transfection Reagent (Qiagen, Germany) is based on activated dendrimer technology for a DNA transfection reagent. In the case of imaging fields, a new dendritic magnetic resonance contrast agent was investigated and launched to the market under the name of Gadomer-17® by Bayer/Schering Pharma AG, Germany. Using PAMAM as a scaffold/material, Siemens (Germany) successfully developed cardiac diagnostics of Stratus®, EMD Merck (USA) generated siRNA delivery vector of PrioFect®. Besides, other applications of protein detection amplifiers such as UltraAmp^TM^, Affymetrix/Genisphere, (Inc., USA), surgical adhesives such as Dendri-Lens™ /OcuSeal™ (HyperBranch Medical Technology, Inc., USA) were also carried out to launch to the market completely. However, those applications of dendrimers have only stopped at the simple stages by exploiting the surface functional groups or nanoscale dimensions [13]. In the future, advanced drug delivery, especially in anti-cancer drug loading, should be investigated for clinical trials as well as for FDA approval.

In our previous studies, unmodified PAMAM dendrimers were successfully synthesized for the loading and releasing of cisplatin, a first generation platinum-based drug. The size of the system was well-controlled but too small (3–8 nm), resulting in very low drug loading capacity [19,22]. Even though the dendrimer’s size was larger at higher generations, the higher toxicity and lesser stability limit their use [23]. Therefore, surface modification of low generation PAMAM with polyethylene glycol (PEG), a biocompatible and hydrophilic polymer, is a better approach for drug loading augmentation since conjugated PEG can drastically increase the cavity space of PAMAM [17,24]. In addition, non-specific interaction between PAMAM dendrimer and serum proteins was also averted by PEG, thus preventing the uptake by the reticuloendothelial system (RES), reducing the renal clearance and prolonging the circulation time of PAMAM in the bloodstream [12,25,26,27,28]. More importantly, the presence of PEG on the surface of PAMAM helps overcome the charge-associated cytotoxicity of PAMAM dendrimer (cell lysis) by deterring the electrostatic interaction between positive charged amino groups of PAMAM’s periphery and biological membrane [29,30]. These advantages of PEG-modified PAMAM dendrimers were confirmed in the literature. For example, Kumar D. and co-workers prepared PEGylated PAMAM dendrimers for prolonged delivery of anti-HIV drug. Results revealed that the entrapped drug in PEGylated PAMAM dendrimer was higher than that of naked PAMAM dendrimer [31]. In terms of toxicity reduction, Li Y. et al. proved that substitution of amino surface groups of PAMAM G4.0 with PEG can remarkably reduce its cytotoxicity [32]. A similar phenomenon can also be seen in the study of D’Emanuele A. et al. [33]. 

**Figure 1:**
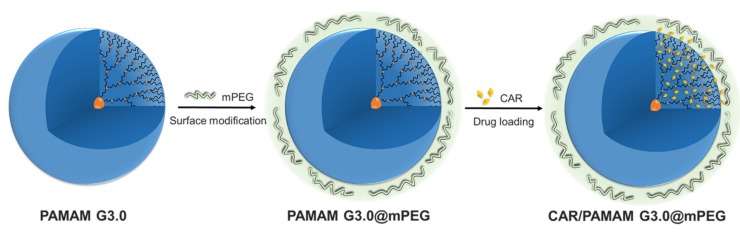
Illustration showing the surface modification of low generation polyamidoamine G3.0 dendrimer (PAMAM G3.0) with methoxypolyethylene glycol (mPEG) and the loading of carboplatin (CAR) into PAMAM G3.0@mPEG to form CAR/PAMAM G3.0@mPEG.

In this study, low generation PAMAM G3.0 dendrimer (PAMAM G3.0) was synthesized and partially surface modified with methoxypolyethylene glycol (mPEG) (Figure 1) for CAR delivery, to improve the limitations related to size and loading capacity of previous CAR-loaded DDSs. Chemical structure, size and morphology of the obtained products were characterized by proton nuclear magnetic resonance (^1^H-NMR), Fourier transform infrared spectroscopy (FT-IR), transmission electron microscopy (TEM), and Gel permeation chromatography (GPC). Zeta potential and biocompatibility were examined to confirm the reduction of charge-related cytotoxicity of modified PAMAM dendrimer. CAR loading capacity was evaluated. Also, the anticancer efficacy of CAR-loaded mPEG-modified PAMAM G3.0 was tested on HeLa, A549 and MCF7 cell lines using cytotoxicity assay. The obtained results give insights into the potential of PAMAM G3.0@mPEG dendrimer as an efficient delivery system for the delivery of a drug that has strong side effects and fast renal clearance like CAR, which could be a promising approach for cancer treatment.

## 2. Materials and Methods 

### 2.1. Materials

Ethylenediamine (EDA, Mw 60.10 Da) and toluene were purchased from Merck (Darmstadt, Germany). Methyl acrylate (MA, Mw 86.09 Da), poly (ethylene glycol) methyl ether (mPEG, Mw: 5 kDa), 4-Nitrophenyl chloroformate (NPC, Mw 201.56 Da), tetrahydrofuran (THF), and diethyl ether were purchased from Sigma-Aldrich (St. Louis, MO, USA). Methanol was supplied by Fisher Scientific (Waltham, MA, USA). Carboplatin (CAR) was received from TCI (Tokyo, Japan). Spectra/Por® Dialysis Membrane (MWCO 3.5 kDa and 12–14 kDa) was purchased from Spectrum Laboratories, Inc. (Rancho Dominguez, CA, USA). All reagents and solvents were used as received without further purification.

### 2.2. Preparation of PAMAM G3.0 Dendrimer

The preparation of PAMAM G3.0 dendrimer started from the EDA initiator core followed by the attachment of branches utilizing two consecutive chain-forming reactions, the exhaustive Michael addition reaction and the exhaustive amidation reaction, as reported in previous work [34]. Initially, 20 mL of EDA was added dropwise into 150 mL of MA dissolved in methanol. The mixture was kept under stirring at 0°C for 3 h and then at room temperature for 2 days. During the reaction, primary amine groups of the EDA core reacted with an excessive amount of acrylate groups of MA via Michael addition reaction to form tetraester (G-0.5). Methanol and unreacted MA were removed by rotary vacuum evaporator (Strike 300, Lancashire, PR6 0RA, UK). Thereafter, 20 g of G-0.5 dissolved in methanol was added dropwise to 130 mL of EDA at 0°C with stirring for 3 h. The amidation reaction between methyl propionate groups of G-0.5 and amine of EDA was carried out for 4 days at room temperature, generating PAMAM G0.0. The solution was rotated under vacuum at 45°C in a mixture of toluene and methanol (9:1 *v/v*) to remove excess EDA, followed by dialysis (dialysis membrane MWCO 3.5 kDa) against methanol. Finally, a rotary vacuum evaporator was used for the removal of methanol. Alternative repetition of these reactions leads to the higher generation of dendrimer, in which half-generation PAMAM was obtained after each addition reaction while full generation PAMAM was obtained after each amidation reaction. Generally, PAMAM G3.0 with 32 amine groups was achieved after four times of amidation reaction. By measuring the mass of purified G3.0, we calculated a yield of PAMAM G3.0 that was 89%.

### 2.3. Preparation of Activated mPEG-NPC 

Activated mPEG was prepared following our previous literature with minor modifications [35]. Briefly, a mixture of mPEG (10.0 g, 2 mmol) and NPC (0.4837 g, 2.4 mmol), in a three-neck flask, was melted into a paste at 65°C and stirred under vacuum conditions for 6 h. The paste was allowed to cool down to 40°C before adding 15 mL of THF to solubilize the mixture. The reaction was carried out at room temperature for 12 h. After that, the resulting solution was slowly added to diethyl ether for the precipitation. The precipitate was then filtered and dried under vacuum to obtain NPC-mPEG in the form of white powder. 

### 2.4. Preparation of mPEG-Modified PAMAM G3.0 Dendrimer (PAMAM G3.0@mPEG)

To obtain PAMAM G3.0@mPEG, 0.448 g of mPEG-NPC was dissolved in distilled water and slowly added to PAMAM G3.0 solution in dropwise manner. The reaction was kept under constant stirring at room temperature for 48 h. Then, the resulting solution was dialyzed against distilled water for 4 days using dialysis membrane of MWCO 12–14 kDa. Freeze-drying was performed to give the product in powder form. 

### 2.5. Preparation of CAR/PAMAM G3.0@mPEG 

CAR (10 mg) was added to methanol and stirred for 1 h to make sure that the drug was completely dissolved prior to being added in dropwise manner into 10 mL of PAMAM G3.0@mPEG solution. The mixture was stirred for 24 h at room temperature, followed by the removal of methanol under vacuum. The product was purified by dialysis against deionized water for 1 h (MWCO 3.5 kDa) and then freeze-dried to obtain CAR/PAMAM G3.0@mPEG. The solution outside dialysis membrane containing free CAR was collected for the determination of loaded drug later. The yield of mPEGylation G3.0 synthesis was over 80%.

### 2.6. Characterizations 

The chemical structures of products were examined by ^1^H-NMR (Bruker Advance 500 MHz, Bruker, Billerica, MA, US) and FT-IR spectroscopy (FT-IR/NIR Spectroscopy Frontier, Perkin Elmer, Waltham, MA, US). Samples were mixed with KBr and pressed (800 MPa) into a pellet. Gel permeation chromatography (GPC, Agilent GPC/SEC 1100, Santa Clara, CA, USA) was used to measure the molecular weights of G3.0 and G3.0@mPEG. NaNO_3_ aqueous solution of 0.1 M was the mobile phase. The applied column was 120 Ultrahydrogel (Waters, Milford, MA, USA). The flow rate of the mobile phase passing column was 1 mL/min. The sample volume of 100 μL was injected manually. The detector and column temperature were 35°C. Various PEG standards (Waters) were used to determine the MW. The measurement acquired in a range of 4000–500 cm^−1^ with a resolution of 4 cm^−1^. Size and morphology of the particles were imaged by TEM (JEM-1400, Japan) at an accelerating voltage of 100 kV. Zeta potential of the samples was measured by zeta potential analyzer (SZ-100, Horiba, Japan). Sample dispersions (1 mg/mL) were sonicated for 5 min and stabilize for at least 60 min before the measurement. The average zeta potential of each sample was obtained from 3 test runs. 

### 2.7. CAR Loading Capacity and In Vitro CAR Release

The drug entrapment efficiency of CAR/PAMAM G3.0@mPEG was quantified using a high performance liquid chromatography (HPLC) system (HPLC PerkinElmer Flexar, Japan) (*n* = 3). Drug entrapment efficiency (%) was the weight ratio of CAR in the particles and fed CAR, in which the weight of CAR in particles was calculated indirectly from the free CAR collected outside the dialysis bag. 

The in vitro release behavior of CAR was determined using the dialysis method. Briefly, 1 mL of CAR/PAMAM G3.0@mPEG (CAR content, 0.2 mg/mL) suspended in PBS (0.01 M, pH 7.4) were transferred to a dialysis bag (MWCO 3.5 kDa) and immersed into a vial containing 14 mL of medium. The vials were placed in an orbital shaker bath that can both maintain the temperature of 37°C and the continuously shaken at 100 rpm. At predetermined time intervals, the release medium (14 mL) was collected, filtered (pore size = 0.22 µm), and replaced with an equal volume of fresh media. The collected media were finally lyophilized and used to determine the release amount of CAR by HPLC. The measurements were run in triplicate. 

### 2.8. Cell Viability Assays

Biocompatibility of the materials was assessed by both live/dead assay and Resazurin assay against mouse fibroblast cells L929. For live/dead assay, cells were cultured in Dulbecco’s Modified Eagle’s medium (DMEM, Gibco, Invitrogen) supplemented with 10% FBS and 1% penicillin/streptomycin at 37°C, 5% CO_2_, and humidified condition. In a dark room, the media were removed and replace with 25 µL of fluorescein diacetate (FDA) (10 mM) and ethidium bromide (EB) (7.5 mM). After a brief incubation for 3 min, cells were rinsed several times with PBS and finally examined under microscope (Eclipse Ti-E Inverted Microscope System, Nikon, Japan) equipped with a digital camera. 

Cytotoxicity of synthesized products on HeLa, A549, and MCF7 cell lines was determined by standard Resazurin assay. Briefly, cells were seeded in a 96-well plate at a density of 1.5 × 10^5^ cells/well and cultured for 24 h. Then, the media were withdrawn and replaced with fresh media containing free CAR or CAR/PAMAM G3.0@mPEG with equivalent amount of loaded CAR. Wells containing cells treated with medium only were used as control and assigned to 100% survival. After 48 h of incubation, the solution from each well was discarded, followed by washing the cells twice with 1X PBS. Thereafter, Resazurin solution (10 µL, 0.2 mg/mL) was added. The cells were further incubated for 4 h. The fluorescent signal was monitored at Ex/Em 560/590 nm using a micro-plate reader (Varioskan™ LUX multimode, Thermo Scientific). The percentage of cell viability was calculated by normalizing the fluorescence intensity of samples to that of control group, presented by Equation (1):

(1) $$\mathrm{Cell}\;\mathrm{viability}\;\left(\%\right)\;=\frac{\left({\left[\mathrm{Abs}\right]}_{\mathrm{sample}}-{\left[\mathrm{Abs}\right]}_{\mathrm{blank}}\right)}{\left({\left[\mathrm{Abs}\right]}_{\mathrm{control}}-{\left[\mathrm{Abs}\right]}_{\mathrm{blank}}\right)}\;\times \;100$$

## 3. Results and discussion

### 3.1. Preparation of PAMAM G3.0@mPEG

**Figure 2:**
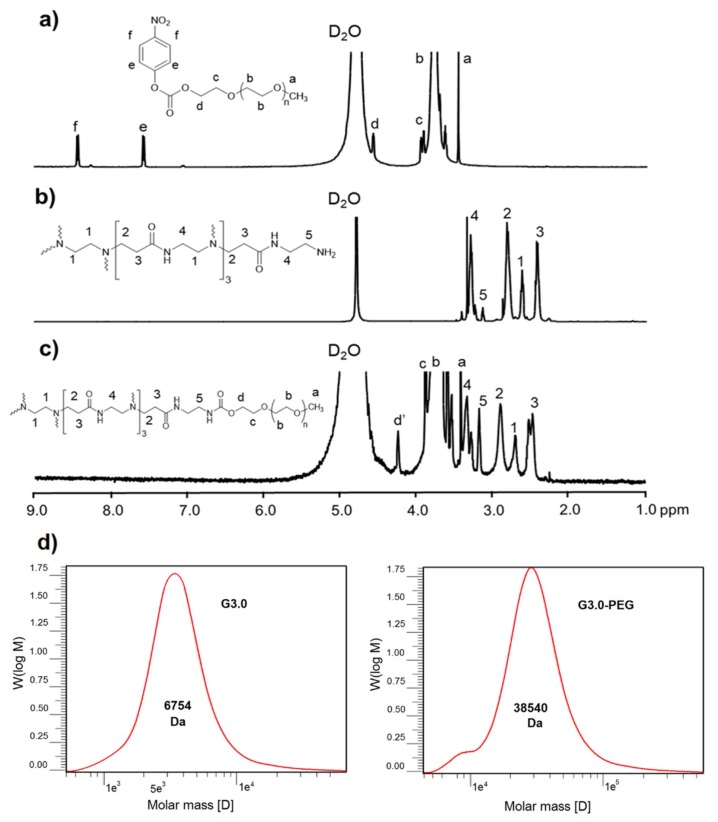
Proton nuclear magnetic resonance (^1^H-NMR) (D_2_O, δ in ppm) spectra of (**a**) mPEG-NPC, (**b**) PAMAM G3.0, (**c**) PAMAM G3.0@mPEG, and (**d**) Gel permeation chromatography (GPC) chromatogram of G3.0 and G3.0@mPEG.

Herein, mPEG having only one terminal hydroxyl group was utilized to avoid the loops and cross-links formation on the periphery of PAMAM dendrimer. To achieve an efficient conjugation between mPEG and PAMAM G3.0, the hydroxyl group of mPEG was activated by coupling reagent NPC to form active intermediates (mPEG-NPC) that can readily react with G3.0’s amine groups via stable urethane linkages. ^1^H-NMR and FT-IR spectroscopy were used to characterize the chemical structures of obtained products. Figure 2a–c revealed ^1^H-NMR spectra of mPEG-NPC, PAMAM G3.0, and PAMAM G3.0@mPEG, respectively. As shown in Figure 2a, several resonance signals at 2.57–2.68 ppm (peak 1), 2.73–2.93 ppm (peak 2), 2.32–2.52 ppm (peak 3), 3.25–3.33 ppm (peak 4), and 3.09–3.18 ppm (peak 5) were respectively assigned to methylene protons of -CH_2_-N<, -CH_2_-NH-, -CH_2_-CO-NH-, -CO-NH-CH_2_-, and -CH_2_-NH_2_- groups. The presence of these signals indicated the successful preparation of PAMAM G3.0 dendrimer. In Figure 2b, peaks appeared at 3.46 ppm (peak a) and 3.76–4.57 ppm (peak b, c, d) and were attributed to methyl ended groups –OCH_3_ and protons in repeat units of mPEG. Besides, doublet at 7.58 ppm (peak e) and 8.45 ppm (peak f) corresponding to two typical signals of NPC in activated mPEG were also detected. In addition to resonance signals indicating the presence of both PAMAM G3.0 dendrimer and mPEG, ^1^H-NMR spectra of PAMAM G3.0@mPEG (Figure 2c) showed a complete absence of NPC’s signals and a chemical shift of protons Hd’ from 4.57 ppm to 4.27 ppm (NHCOO-CH_2_-CH-) to confirm the formation of PAMAM G3.0@mPEG. This could be explained by the substitution of NPC by urethane linkage to covalent link mPEG onto PAMAM G3.0’s surface. 

Since PAMAM dendrimer possesses a symmetric structure in which every proton in the molecules has a theoretically identifying number, ratios of protons in PAMAM structure are equal to the integral ratios of resonance signals in the ^1^H-NMR spectrum [36]. According to this property, the actual number of mPEG conjugated on PAMAM G3.0 can be calculated using Equation (2) based on the integral ratio of Hd’ signal (δH = 4.27 ppm) and H3 (δH = 2.44–2.59 ppm) in mPEGylated PAMAM G3.0 spectrum. Proton Hd’ represented the total linkage between mPEG and PAMAM while proton Hi acts as representative of PAMAM G3.0. As a result, there were about 7.78 conjugated mPEG moieties per PAMAM G3.0 molecules.

(2) $$\mathrm{Number}\;\mathrm{of}\;\mathrm{conjugated}\;\mathrm{mPEG}\;=\;\frac{\mathrm{Sum}\;\mathrm{of}\;\mathrm{proton}\;\mathrm{at}\;\mathrm{position}\;(3)\;\times \;\int^{\text{​}}H\;(d\prime )}{\mathrm{Sum}\;\mathrm{of}\;\mathrm{proton}\;\mathrm{at}\;\mathrm{position}\;(d\prime )\;\times \;\int^{\text{​}}H\;(3)}$$

In which

(3) $$\int^{\text{​}}H\;\left(d\prime \right)\;:\;\mathrm{Integral}\;\mathrm{of}\;\mathrm{proton}\;\mathrm{at}\;\mathrm{position}\;(d\prime )$$

(4) $$\int^{\text{​}}H\;\left(3\right)\;:\;\mathrm{Integral}\;\mathrm{of}\;\mathrm{proton}\;\mathrm{at}\;\mathrm{position}\;(3)$$

Figure 2d showed the *M_W_* of G3.0 and G3.0@mPEG determined from GPC technique were 6754 Da (symbolized as $M_{W}^{G3.0\left(GPC\right)}$) and 38,540 Da (symbolized as $M_{W}^{G3.0@mPEG\left(GPC\right)}$) respectively. To find the number of conjugated mPEG ($M_{W}^{mPEG}$ = 4000 Da), the formula was established as following:

(5) $$\text{Number of conjugated mPEG}=\frac{M_{W}^{G3.0@mPEG\left(GPC\right)}-M_{W}^{G3.0\left(GPC\right)}}{M_{W}^{mPEG}}$$

The *M_W_* of G3.0 found by GPC was an approximate value compared to the theoretical value of 6909 Da. This result could confirm the successful synthesis of G3.0 along with the ^1^H-NMR spectrum. By using Equation (3), the number of conjugated mPEG was 7.95. This value agreed with the result of Equation (2). One more time, the mPEGylation of G3.0 was confirmed, also the mPEG moieties were calculated relatively. In addition, the single peak was recognized in GPC techniques for each polymer which revealed the high purity of G3.0 and G3.0@mPEG.

**Figure 3:**
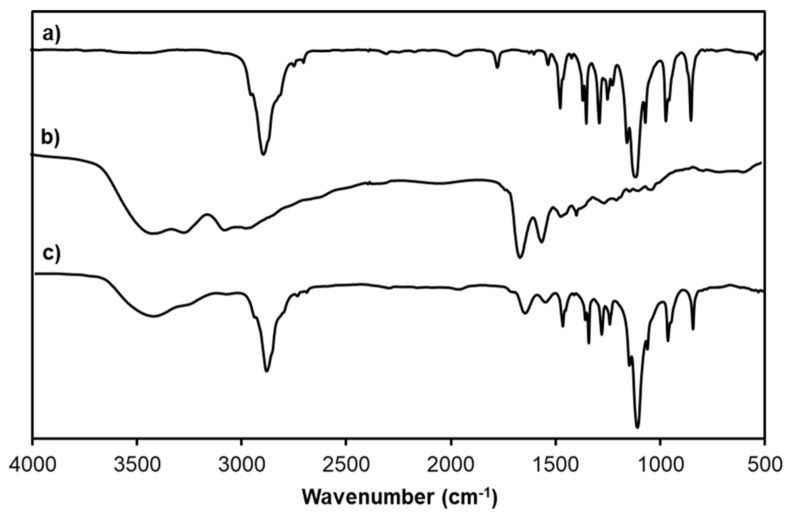
Fourier transform infrared spectroscopy (FT-IR) patterns of (**a**) mPEG-NPC, (**b**) PAMAM G3.0, and (**c**) PAMAM G3.0@mPEG.

Furthermore, the successful modification of PAMAM G3.0 with mPEG was also evaluated by FT-IR analysis. FT-IR patterns of mPEG-NPC, PAMAM G3.0, and PAMAM G3.0@mPEG are presented in Figure 3. The spectrum of mPEG-NPC reveals distinctive bands at 2888 cm^−1^ (C-H stretching vibration), 1550 cm^−1^ (-NO_2_), and 1111 cm^−1^ (C-O-C). In PAMAM G3.0@mPEG pattern, peaks assigned at 2888 cm^−1^ (C-H), 1108 (C-O-) and 1556 cm^−1^ (N-H bending vibration) for mPEG and PAMAM G3.0, respectively, were detectable. A shift from 1645 to 1630 cm^−1^ attributed to C=O stretching of urethane linkage together with the disappearance of the band at 1550 cm^−1^ (-NO_2_) corresponded to NPC imply that mPEG was covalently linked to amino groups on the surface of PAMAM G3.0. 

### 3.2. Characterization of CAR/PAMAM G3.0@mPEG

**Figure 4:**
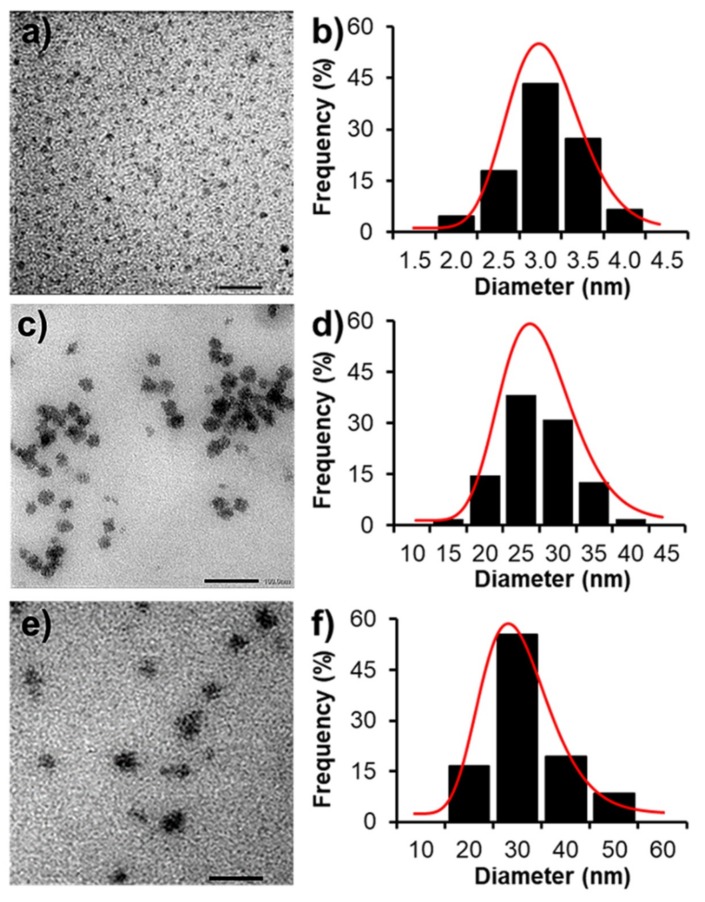
(**a**–**c**) Transmission electron microscope (TEM) micrographs and (**d**–**f**) size distribution of PAMAM G3.0 (scale bar = 20 nm) (**a**,**b**), PAMAM G3.0@mPEG (scale bar = 100 nm) (**c**,**d**), and CAR/PAMAM G3.0@mPEG (scale bar = 100 nm) (**e**,**f**).

Figure 4 shows TEM images and size distribution of unmodified PAMAM G3.0, PAMAM G3.0@mPEG, and CAR/PAMAM G3.0@mPEG. In comparison to PAMAM G3.0 (5.9 ± 1.1 nm), there was a remarkable increment in the particle size of PAMAM G3.0@mPEG (32.6 ± 0.9 nm). This phenomenon indicates the presence of mPEG chains on the surface of PAMAM G3.0, further confirming the success in PAMAM G3.0@mPEG preparation. The water-soluble and non-ionic PEG chains modified on G3.0 surfaces were to increase the hydration level of nanoparticles to avoid the adsorption of opsonins (serum proteins) which attracts the phagocytes [37]. Therefore, the PEGylated G3.0 could hide from the macrophages to prolong their blood circulation time. In addition, PEGylation caused the enlargement of nanoparticle sizes (Figure 4). This phenomenon leads to formation of steric hindrance, and then reduction of the filtration of globular kidney membrane to extend their circulation time [38]. In fact, a lot of studies have already proved these same advantages of PEGylation [39,40,41].

After drug loading, the size of CAR/PAMAM G3.0@mPEG was evidently recognized in the range of 20–50 nm, which is more favorable for the delivery of CAR than unmodified PAMAM with really small size or other previous CAR-loaded nanoparticles (liposomes, PCL nanoparticles, carbon-based nanocontainers) with a larger size range (~244–311 nm up to 10–30 µm) [9,10,11]. It was reported that DDSs having a size of less than 10 nm will be quickly cleared from the circulatory system due to extravasation and those with the size larger than 200 nm tend to be accumulated in the spleen or taken up rapidly by the mononuclear phagocyte system (MPS). By contrast, nanoparticles that range from 10–200 nm in size can be easily entrapped by endosome during endocytosis, thus facilitating the passive targeting and accumulation of nanoparticles to tumors through EPR effect and reducing toxicity to normal cells [26,42,43,44,45]. Moreover, the conjugation of hydrophilic mPEG on PAMAM G3.0 surface also avoids the action of RES and reduce renal clearance [25,46]. Therefore, PAMAM G3.0@mPEG with a desirable size range (~30–50 nm) after surface modification and drug loading serves as promising nanocarrier to prolong the fate of CAR in blood circulation and to effectively deliver CAR to tumor cells.

In cancer treatment, the capacity of a DDS to effectively encapsulate chemotherapeutic agents is very important to improve the therapeutic index of the loaded drug and the therapeutic efficacy of the system [26,47]. Our previous studies have demonstrated that, in spite of exhibiting less toxicity compared with higher generation PAMAM dendrimer, the small size and open structure of low generation (G2.5-G3.5) cannot entrap the drug inside well, resulting in low drug entrapment, particularly only 26.64–27.83% for the first generation platinum-based drug cisplatin [19,22]. In another study, PAMAM G3.5 was developed for CAR delivery to treat murine retinoblastoma. The formulation showed positive results; however, the efficacy of CAR loading was limited to 47.54% [48]. In this present work, by surface functionalizing low generation PAMAM with mPEG, it was highly expected that the amount of entrapped drug could be efficaciously enhanced. mPEG chains attached on the PAMAM G3.0’s periphery acquired the extended conformation through the repulsion between the adjacent chains, which makes the internal space of the dendrimer remarkable larger. It was reported that the size of PEGylated dendrimers was way more spacious than the total of PEG and dendrimer alone [14]. For PAMAM G3.0@mPEG, it was already examined in the above section that the increment in size could be up to 26.7 nm. As expected, PAMAM G3.0@mPEG could entrap up to 83.98 ± 0.24% *wt/wt* of CAR. 

### 3.3. Charge-Related Cytotoxicity 

**Figure 5:**
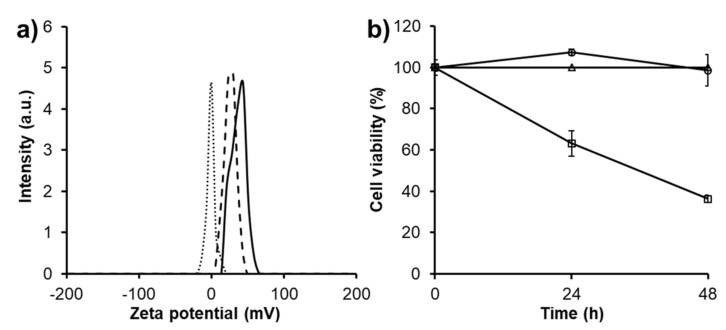
(**a**) Zeta potential showing the surface charge of PAMAM G3.0 (solid curve), PAMAM G3.0@mPEG (dashed curve), and CAR/PAMAM G3.0@mPEG (square dotted curve), (**b**) Viability of L929 mouse fibroblast cells after 48 h cultured with media (triangle), media containing PAMAM G3.0 (500 μg/mL) (square), and media containing PAMAM G3.0@mPEG (500 μg/mL) (circle).

Because of the positive charged amino groups on PAMAM’s periphery directly contributed to the non-specific toxicity of unmodified PAMAM, zeta potential of modified PAMAM G3.0 dendrimers was evaluated [29,34]. As displayed in Figure 5a, both PAMAM G3.0@mPEG and CAR/PAMAM G3.0@mPEG showed significant reduced zeta potential (26.3 ± 0.44 mV and 0.20 ± 0.17 mV) as compared to unmodified PAMAM G3.0 (44.63 ± 0.31 mV). This revealed that surface modification with mPEG can effectively eliminate the strongly positive charge of PAMAM G3.0. For CAR/PAMAM G3.0@mPEG, the charge was further neutralized after drug loading thanks to a greater shielding effect when mPEG chains wrap around loaded CAR.

The reduction in charge-related cytotoxicity of PAMAM G3.0 by mPEG surface modification, as a result of its decreased positive surface charge, was examined on L929 fibroblast cells using resazurin assay (Figure 5b). PAMAM G3.0 showed time-dependent cytotoxicity to fibroblast cells, which causes 36.8% and 63.65% cell death after 24 h and 48 h, respectively. For PAMAM G3.0@mPEG, it was basically non-toxic even at high concentrations (up to 500 µg/mL), with the cell viability about 98.59% after culturing for 48 h. This result implied that the cytotoxicity of PAMAM G3.0 was nearly eliminated within tested concentration range (≤ 500 µg/mL) after partially surface modification even though the average conjugated mPEG moieties per PAMAM G3.0 dendrimer was only 7.78. A similar phenomenon was observed in a study of A. D’Emanuele et al., in which above 90% of cells were alive after being treated with a low concentration (< 0.01 mM) of PAMAM G4.0 modified with 4 PEG chains [33].

Also, the amine groups of PAMAM have been considered a major reason for hemolysis [12]. The higher amine content, depending on the concentration and/or even generation of PAMAM dendrimers, will cause stronger hemolysis. In the case of G3.0 PAMAM, Domanski et al. reported that 77 µM could cause 50% of released hemoglobin [49,50]. Therefore, the positive charge of G3.0 surface had to be decreased to overcome the hemolytic phenomenon [12]. Herein, G3.0 PAMAM dendrimers were modified about 24.3 % of positive charge. Moreover, the long PEG chains could also help to partially hide the remaining amine groups. Zhu et al. reported that the hemolysis of PEGylated PAMAM was less than 5%. Singh et al. also demonstrated the hemolytic percentage of folic acid-PEG-PAMAM being less than 5% which corresponded with many other studies [51,52]. Considering the good cell viability results and referring to the hemolysis results of PEGylated G3.0, it was found that the PEGylated low PAMAM generation of G3.0 being non-toxic at tested concentrations.

**Figure 6:**
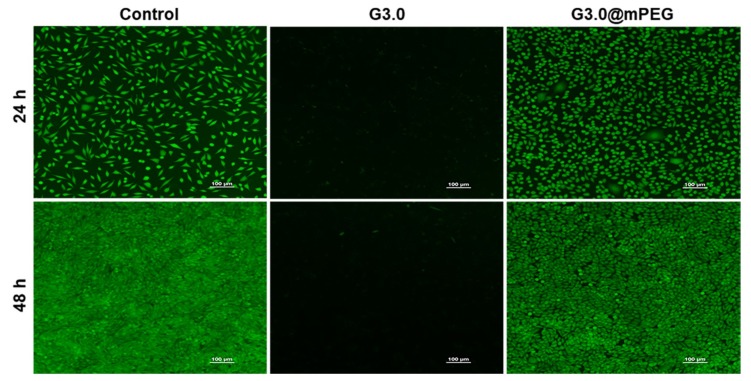
Fluorescent image of live/dead staining of L929 cells after culturing for 24 h and 48 h with media (control), PAMAM G3.0 (500 µg/mL) and PAMAM G3.0@mPEG (500 µg/mL). Scale bar = 100 µm.

The morphology of L929 cells incubated with PAMAM G3.0 and PAMAM G3.0@mPEG for 24 h and 48 h is further visualized in Figure 6. As compared to the control, PAMAM G3.0@mPEG showed a negligible toxic effect to L929 cells. Approximately 98% of cells were viable and well proliferated after long incubation time (24 h and 48 h) with a high concentration of PAMAM G3.0@mPEG (500 µg/mL). On the contrary, unmodified PAMAM G3.0 exhibited dramatic cytotoxicity to the cells, which was also observed in other work [53]. The fluorescent images of cell morphology were closely consistent with the resazurin result, in which the discrepancy may be due to the differences in assay principle. These results indicated that PAMAM G3.0 dendrimer modified mPEG overcomes its charge-associated cytotoxicity, paving the way for being applied as DDS in cancer treatment with great cytocompatibility. 

### 3.4. Release Behavior of CAR 

**Figure 7:**
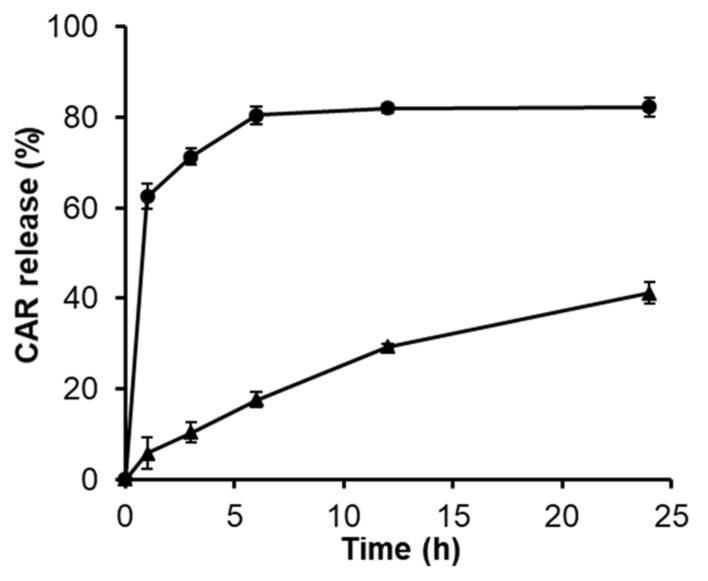
Release profiles of CAR from PAMAM G3.0@mPEG (triangle). The release of free CAR with equal amount to that loaded in PAMAM G3.0@mPEG was carried out as a control (round).

In vitro release behavior of CAR from PAMAM G3.0@mPEG was evaluated (Figure 7). The release of free CAR with an equivalent amount to CAR that entrapped in PAMAM G3.0@mPEG was performed as a control. From the dialysis membrane, free CAR showed an initial burst phenomenon with an amount accounting for 62.49% of loaded drug within the first hour whereas PAMAM G3.0@mPEG can maintain merely 94% of CAR in its dendritic structure in the same time interval. After 6 h, nearly complete free CAR (80.55%) was released; however, only 17.66% loaded CAR was cumulatively detected in the medium. Thereafter, CAR continuously released from PAMAM G3.0@mPEG in a controlled and sustained manner and reached 41.23% released at 24 h, which is just half of the released free CAR. This behavior is beneficial for the delivery of the drug to the target site with minimum loss, especially with the drug that has strong side effects and fast renal clearance like CAR (90% of administered dose is excreted within 24 h) [8]. In addition, PEG is well known as a cilia molecular. Thus, nanoparticles modified PEG chains contained flexible tails that can insert into the mucus layer through a diffusion mechanism. Mucus is a sticky gel coating on the cell membranes to eliminate foreign factors. The PEG chains covering PEGylated nanoparticles support them to easily penetrate through the mucus layer and accumulate in cells. Then the loaded drugs will be released locally at a high amount [54]. For all results, a sustainable and accumulative amount of loaded drugs will be achieved for PEGylated G3.0 formula.

### 3.5. In Vitro Activities of CAR/PAMAM G3.0@mPEG against Various Types of Cancer Cells 

**Figure 8:**
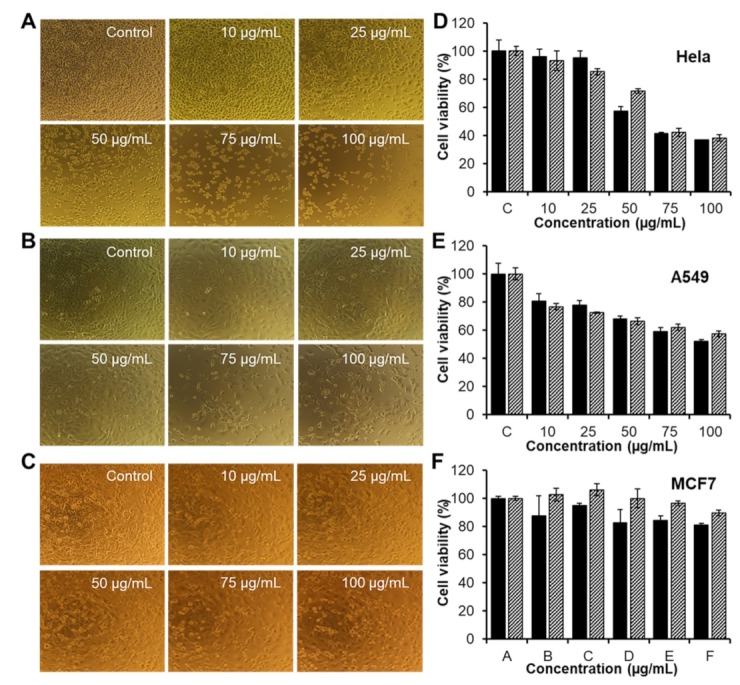
(**A**) Image of Hela cells, (**B**) A549 cells, and (**C**) MCF7 cells incubated with free CAR and CAR/PAMAM G3.0@mPEG at different concentration observed under microscope for 48 h. (**D**) Viability of HeLa cells, (**E**) A549 cells, and (**F**) MCF7 cells incubated with free CAR (black) and CAR/PAMAM G3.0@mPEG (diagonal) at various doses (10–100 µg/mL) for 48 h, determined by Resazurin assay. Data are expressed as mean ± SD (*n* = 3).

The anticancer activities of free CAR and CAR/PAMAM G3.0@mPEG, containing an equivalent amount of CAR, were further studied in HeLa, A549, and MCF7 cell lines using standard Resazurin assay (Figure 8A–C). Cells treated with medium only were used as the control and assigned to 100% survival. As shown in Figure 8D–F, the anticancer effects of free CAR and CAR/PAMAM G3.0@mPEG were dependent on cell types and the therapeutic dose. By treating with an increased concentration of samples (10–100 μg/mL), a corresponding decrease in the cell viability of all samples was observed in three cell lines after 48 h of incubation. For HeLa cells (Figure 8B), free CAR at 50 μg/mL caused severe cytotoxicity leading to nearly 50% of tumor cells being killed, whilst CAR/PAMAM G3.0@mPEG started to exert their anticancer activity and made the cell viability drop to around 71.43% at the same concentration. At higher concentrations (75–100 μg/mL), CAR/PAMAM G3.0@mPEG was as active as free CAR. Similarly, the difference between inhibition degree of A549 cell proliferation of CAR/PAMAM G3.0@mPEG and that of free CAR at all tested concentrations was not much (Figure 8E), considering that PAMAM G3.0@mPEG nanocarriers were cytocompatible to the cells. Interestingly, HeLa cells were more sensitive than A549 and MCF7 cells, upon being given the same dose of samples (free CAR and encapsulated CAR), especially at higher doses. At 100 µg/mL, only 19.1% and 10.3% of MCF7 cells were respectively killed by free CAR and CAR/PAMAM G3.0@mPEG while that of HeLa cells were 63.4% and 61.8%. Taken together, these results indicated that CAR/PAMAM G3.0@mPEG could fully retain the bioactivity of released CAR to suppress the proliferation of various types of cancer cells. Further in vitro and in vivo research is recommended to obtain more details about the therapeutic efficacy of developed CAR/PAMAM G3.0@mPEG formulations.

## 4. Conclusions

A partial surface modification of PAMAM G3.0 dendrimer with mPEG to form PAMAM G3.0@mPEG as a promising platform for CAR delivery has been developed. The resulting PAMAM G3.0@mPEG has successfully improved the drawback related to the size and loading capacity of previous CAR-loaded DDSs, having a desirable size range (~30–50 nm) and high entrapment efficiency. The release profile of CAR/PAMAM G3.0@mPEG showed controlled and sustained release without any initial burst phenomenon. Additionally, cell viability assays revealed that PAMAM G3.0@mPEG could be safely used and fully retain the anticancer activity of released CAR to suppress the proliferation of various cancer cell lines.
